# Family-based associations in measures of psychological distress and quality of life in a cardiac screening clinic for inheritable cardiac diseases: a cross-sectional study

**DOI:** 10.1186/1471-2350-14-1

**Published:** 2013-01-08

**Authors:** Catherine McGorrian, Charlene McShane, Colin McQuade, Ted Keelan, Jim O Neill, Joseph Galvin, Kevin Malone, Niall G Mahon, Mary Codd

**Affiliations:** 1 The Heart House, Mater Misericordiae University Hospital, Dublin, 7, Ireland; 2 UCD School of Public Health, Physiotherapy and Population Science, University College Dublin, Dublin, Ireland; 3 Department of Cardiology, Connolly Hospital, Blanchardstown, Dublin, 15, Ireland; 4 Department of Psychiatry and Mental Health Research, St Vincent’s University Hospital, Dublin, 4, Ireland

**Keywords:** Screening, Inherited cardiac diseases, Channelopathy, Sudden cardiac death, Sudden arrhythmic death syndrome, Anxiety, Depression, Family-based evaluation

## Abstract

**Background:**

Family-based cardiac screening programmes for persons at risk for genetic cardiac diseases are now recommended. However, the psychological wellbeing and health related quality of life (QoL) of such screened patients is poorly understood, especially in younger patients. We sought to examine wellbeing and QoL in a representative group of adults aged 16 and over in a dedicated family cardiac screening clinic.

**Methods:**

Prospective survey of consecutive consenting patients attending a cardiac screening clinic, over a 12 month period. Data were collected using two health measurement tools: the Short Form 12 (version 2) and the Hospital Anxiety and Depression Scale (HADS), along with baseline demographic and screening visit-related data. The HADS and SF-12v.2 outcomes were compared by age group. Associations with a higher HADS score were examined using logistic regression, with multi-level modelling used to account for the family-based structure of the data.

**Results:**

There was a study response rate of 86.6%, with n=334 patients providing valid HADS data (valid response rate 79.5%), and data on n=316 retained for analysis. One-fifth of patients were aged under 25 (n=61). Younger patients were less likely than older to describe significant depression on their HADS scale (p<0.0001), although there were overall no difference between the prevalence of a significant HADS score between the younger and older age groups (18.0% vs 20.0%, p=0.73). Significant positive associates of a higher HADS score were having lower educational attainment, being single or separated, and being closely related to the family proband. Between-family variance in anxiety and depression scores was greater than within-family variance.

**Conclusions:**

High levels of anxiety were seen amongst patients attending a family-based cardiac screening clinic.Younger patients also had high rates of clinically significant anxiety. Higher levels of anxiety and depression tends to run in families, and this has implications for family screening and intervention programmes.

## Background

A number of cardiac conditions which can cause cardiac arrhythmia and even sudden cardiac death (SCD) are now known to be of genetic etiology. Tagetted, family-based clinical and genetic cardiac screening for at-risk families is now recommended by many agencies, with the aim of early identification of potentially dangerous cardiac diseases [1]. These diseases include certain cardiomyopathies as well as the cardiac “ion channelopathy” disorders. In the majority of cases, these conditions are inherited in an autosomal dominant manner [2].

In cases where a person dies suddenly and unexpectedly, with no pathological findings at autopsy examination to explain the death, the event is termed Sudden Arrhythmic Death Syndrome, or SADS [3]. Previous studies have shown that in up to half of families with a SADS bereavement, an inherited pro-arrhythmic cardiac condition can be identified in living family members [4-8]. Appropriate and timely management of these cardiac conditions can reduce the risk of a sudden death [9,10]. Family cardiac screening clinics perform protocol-driven clinical cardiac screening both in families with a known history of an inheritable cardiac disease, and in families with a SADS bereavements [11].

One feature of SADS death is the unexpectedness of the bereavement. This may result in a more difficult grief reaction [12]. There can also be fearfullness for the physical health and wellbeing of other family members, and the anxiety for parents in particular can be very great [13]. In our clinic, we have observed that parents will prioritise family screening in the family’s teenagers and young adults, because of the perceived threat to their health from an undetected cardiac condition. Whilst the psychological status of patients attending for cardiac screening because of an inherited cardiac disease risk has been examined in a number of studies [13-17], there is no specific information on anxiety levels in younger adults coming for such screening, compared with older adults. We sought to define the anxiety and depression burden associated with family-based cardiac screening, to examine whether these traits cluster within families, and to examine the associates of higher levels of anxiety and depression states in this population.

## Methods

### Study population

From September 2010 until September 2011, all persons aged 16 and older, attending for screening at a dedicated family heart screening clinic in a single tertiary cardiology referral centre, were approached for study recruitment. Patients were referred by general practitioner-, specialist- or self-referral, because of either a family history of a known or suspected genetic cardiac disease (typically cardiomyopathy or channelopathy), or because of a family history of SADS. At clinic visit, patients met with a specialist nurse counsellor and underwent protocol-driven clinical cardiac testing with ECG and echocardiogram (for families at risk of cardiomyopathy); ECG, Holter, and Treadmill testing (for families at risk of a channelopathy) and all four tests in families with a history of SADS. Patients then underwent a family-based consultation with a cardiologist with expertise in inherited cardiac diseases, and further assessment with tests such as ajmaline provocation testing or genetic testing were scheduled as indicated [8].

In this study, patients who consented completed a Hospital Anxiety and Depression (HADS) survey [18] and a Short Form-12 version 2 (SF-12v2) survey [19], whilst in the clinic but prior to their clinical assessment. Study data were collected on patient demographic details including age, sex, marital status, education status, whether this was a first or follow-up screening visit, and the indication for screening. Note was made as to whether patients had a family history of a SADS or SCD bereavement, and their relationship to the family index case or “proband”. Patients who were themselves the family proband were excluded from this analysis. All families were attributed a numeric family identifer. Ethical approval was granted by the Mater Misericordiae University Hospital Research Ethics Committee, and patients provided informed consent prior to study entry.

### Study instruments

The study instruments chosen were the SF-12v2 and the HADS. The SF-12 was first devised in 1994 and revised in 1998, and is derived from the longer SF-36, based on items from the Medical Outcomes study. The SF-12v2 yields a physical health component summary (PCS-12) and a mental health component summary (MCS-12), as well as eight subscores in the domains of Physical Functioning (PF), Role-Physical (RP), Bodily Pain (BP), General Health (GH), Vitality (VT), Social Functioning (SF), Role-Emotional (RE) and Mental Health (MH). For version 2 of the SF-12, reliability estimates are quoted for the PCS-12 of 0.89, and the MCS-12 of 0.86 [19].

The HADS score was designed as a screening tool to detect clinically significant anxiety and depression in patients coming to an outpatients setting [20]. It consists of 14 items, seven measuring anxiety and seven measuring depression, and scores for each subscale range from 0 to 21. Mean Cronbach’s alpha for the anxiety subscale (HADS-A) is 0.83, and for the depression subscale (HADS-D) was 0.82 [21]. Cut-point scores for possible “cases” have been suggested at 8 to 10 [20], and for definite “cases”, 11 or more [18,20]. For this study, a cut off of 11 or greater was used to define case status.

### Statistical analysis

Simple descriptive statistics were used to describe the baseline demographic features of the population. With regards to the HADS findings, the anxiety and depression subscales (HADS-A and HADS-D respectively) were calculated. For the SF-12v2 analysis, T scores were derived as described by Ware using the 1998 US norms [19], and the PCS and MCS were described by age in groups. Analysis of variance was used to examine the association of the HADS-A, HADS-D, PCS and MCS with age. As the observations (patients) are nested within families, between- and within-family variance was examined for the subscales. The psychometric properties of the HADS in this population were examined using Cronbach’s alpha to assess internal consistency [22], and the correlations between the HADS and SF-12 scales were examined using a pairwise correlation matrix. Frequencies of endorsing the individual data items in the HADS questionnaire were examined by age group, with comparisons between groups made with the Pearson’s chi square test and the Fisher’s exact test. A p value of <0.05 was accepted as statistically significant.

Logistic regression models were fitted to examine the associations between demographic and patient-specific factors and a high HADS score (score of ≥11 on either subscale, as above). Simple logistic regression was used to examine the individual factors, with adjusted multivariable logistic regression models were fitted which included the covariables which had reached statistical significance at at least the 10% level in the simple models. Multivariable models were fitted both with and without a random effects term to indicate family identification number. All analyses were performed with Intercooled Stata 12 (StataCorp, Texas).

## Results

Of the total population approached, 364 of 420 patients consented and provided data to the study, with 334 respondants providing complete and valid HADS data (response rate of 86.6%, valid response rate 79.5%). A small number of patients (6.6%) came because of a suspected inherited condition in themselves (with no family history) and were excluded from further analysis. Of the study n=316, the mean age was 35.94 years, stadard deviation (SD) 13.03, range 15 to 72, with nearly one-fifth (n=61) patients being aged 24 or younger. Table 1 shows the demographics of the study population. Most patients came with a screening indication of a known family condition (either a channelopathy or cardiomyopathy), with 23.4% presenting because of a history of SADS or sudden infant death syndrome (SIDS). Of the younger patients (age ≤25), over two-fifths attended for cardiomyopathy screening (42.6%, n=26), whereas 27.9% (n=17) attended for channelopathy screening, and 18.3% (n=11) attended for SADS screening. Over half of the younger patient group were female (54.1%, n=33), and 62.3% (n=38) had a family history of SCD or SADS.

**Table 1:** Baseline demographics on n=316 consenting consecutive family heart screening patients with valid psychological wellbeing data

**Variables**	**N=316**
Age in years: Mean (SD)	35.94 (13.03)
Male sex: n(%)	148 (46.8%)
Clinic visit status:	
First visit: n(%)	248 (78.5%)
Second or subsequent visit:n(%)	68 (21.5%)
Education level (of n=300 patients):	
Completed primary school: n(%)	62 (20.7%)
Completed secondary school: n(%)	77 (25.7%)
Completed a secondary diploma: n(%)	75 (25.0%)
Completed a degree: n(%)	86 (28.7%)
Marital status:	
Married or cohabiting: n(%)	131 (41.5%)
Single: n(%)	175 (55.4%)
Widowed or divorced: n(%)	10 (3.2%)
Reason for screening:	
SADS or SIDS family history: n(%)	74 (23.4%)
Cardiomyopathy screening: n(%)	146 (46.2%)
Channelopathy screening: n(%)	74 (23.4%)
Other screening indication: n(%)	22 (7.0%)
Relationship to family index case:	
First degree relative: n(%)	228 (72.2%)
Second degree relative or greater: n(%)	88 (27.8%)
Family History of SCD: n(%)	196 (62.8%)

Internal consistency of the HADS and SF-12 measures were assessed. Cronbach’s alpha for the HADS-A subscale was 0.828, with an average inter-item correlation of 0.411. For the HADS-D subscale, Cronbach’s alpha for the HADS-D subscale was 0.764, with an average inter-item correlation of 0.338. Cronbach’s alpha for the SF items was 0.855, with an average inter-item correlation of 0.349. Pairwise correlations for the HADS-A, HADS-D, PCS and MCS are shown in Table 2, with strong and statistically significant positive correlations between the HADS-A, HADS-D, and MCS.

**Table 2:** Pairwise correlation coefficients for the the key HADS and SF-12v2 subscales

	**HADS-A**	**HADS-D**	**PCS**	**MCS**
HADS-A	r=1.00	-	-	-
HADS-D	r= 0.60; p<0.0001	r=1.00	-	-
PCS	r=−0.10; p=0.08	r= −0.18; p=0.002	r=1.00	-
MCS	r=−0.61; p<0.0001	r= −0.63; p<0.0001	r=− 0.21; p=0.0003	r=1.00

*HADS-A* Hospital anxiety and depression scale anxiety subscale *HADS-D*. Hospital anxiety and depression scale depression subscale *PCS*. Physical health component summary *MCS* Mental health component summary.

Footnote: Where r is the Pearson correlation coefficient.

Key results for both the SF-12 and HADS scores are shown in Table 3, for the whole population and by age subgroups. HADS-D scores increased with age (p<0.0001), indicating increasing prevalence of depressive symptoms, and PCS-12 decreased with age (p=0.01), indicating worsening physical health. Specific comparisons of the HADS measures in the younger age group compared with the older patients are shown in Table 4. Further information on the SF-12v2 subscale measures by sex and by age group is available in Table 5.

**Table 3:** Key HADS and SF-12v2 results, comparing the findings by age group

	**All persons**	**Persons aged ≤ 24**	**Persons aged 25 -39**	**Persons aged 40-54**	**Persons aged 55+**	F statistics, p value*
***Hospital Anxiety and Depression Scale***						
n	316	61	143	75	37	
HADS-A: Mean (SD)	6.87 (3.96)	6.6 (3.94)	7.09 (3.94)	7.24 (3.77)	5.68 (4.33)	F=1.59, p=0.19
HADS-D: Mean (SD)	2.83 (2.70)	1.82 (2.04)	2.46 (2.29)	4.08 (3.09)	3.35 (3.24)	F=10.42, p<0.0001
***Short Form 12 version 2 scale***						
n	294	57	135	70	32	
PCS-12: Mean (SD)	51.62 (8.07)	53.34 (7.04)	52.38 (7.66)	50.26 (8.55)	48.32 (9.33)	F=3.81, p=0.011
MCS-12: Mean (SD)	49.63 (10.51)	50.37 (10.12)	49.41 (9.47)	47.87 (12.28)	53.11 (10.73)	F=1.95, p=0.12

*From an analysis of variance (ANOVA) model with age group (in categories) as the indendent variable; F statistic and p value given for the model as a whole.

*HADS-A* Hospital anxiety and depression scale anxiety subscale *HADS-D* Hospital anxiety and depression scale depression subscale *SD* Standard deviation *PCS-12*. Physical health component summary *MCS-12* Mental health component summary.

**Table 4:** HADS and SF-12v2 results, with specific focus on the younger patients

	**Younger group (age<25) (n=60)**	**Older group (age 25 and over) (n=252)**	**Test statistic, p value**
**HADS-A: Mean (SD)**	6.60 (3.94)	6.93 (3.97)	t=0.57, p=0.56
**HADS-D: Median (IQR)**	1 (3)	2 (4)	z=3.47, p=0.0005
**PCS-12: Mean (SD)**	53.34 (7.04)	51.21 (8.26)	t=−1.80, p=0.074
**MCS-12: Mean (SD)**	50.37 (10.12)	49.46 (10.62)	t=−0.59, p=0.56

*HADS-A* Hospital anxiety and depression scale anxiety subscale *HADS-D* Hospital anxiety and depression scale depression subscale *SD* Standard deviation *PCS-12*. Physical health component summary *MCS-12* Mental health component summary.

**Table 5:** Data table showing means and standard deviations of the sub scales of SF12v2. Scores shown are the mean (standard deviation) of the subscale T scores, normed to 1998 US data

		**PCS**	**MCS**	**PF**	**RP**	**BP**	**GH**	**VT**	**SF**	**RE**	**MH**
**Men**	**Under 25 years (n=27)**	53.60 (6.40)	51.14 (8.34)	52.65 (8.70)	53.25 (6.48)	52.54 (9.10)	52.49 (9.19)	54.45 (7.89)	53.58 (6.15)	49.87 (7.49)	50.99 (9.16)
	**25-39 years (n=72)**	53.10 (7.08)	49.49 (8.21)	51.33 (10.07)	52.75 (7.89)	53.77 (7.04)	50.39 (9.85)	51.99 (9.19)	51.72 (7.73)	50.41 (8.29)	49.70 (9.36)
	**40-54 years (n=21)**	51.98 (7.39)	49.50 (11.93)	52.38 (7.50)	51.52 (7.10)	51.88 (10.77)	46.38 (10.52)	55.07 (9.91)	48.76 (11.21)	49.47 (8.91)	50.13 (10.05)
	**≥55 years (n=17)**	49.72 (6.75)	54.61 (8.48)	48.74 (10.39)	53.30 (5.59)	51.33 (8.37)	48.73 (9.02)	53.28 (8.93)	52.08 (9.31)	50.49 (9.25)	54.18 (8.87)
**Women**	**Under 25 years (n=30)**	53.11 (7.67)	49.68 (11.59)	50.74 (11.09)	53.64 (5.63)	54.16 (8.06)	51.86 (8.50)	52.62 (9.32)	51.20 (8.10)	47.60(10.30)	52.15 (8.40)
	**25-39 years (n=63)**	51.56 (8.25)	49.32 (10.80)	49.86 (9.93)	51.38 (8.09)	52.97 (9.48)	49.95 (8.95)	52.39 (9.26)	48.68 (10.56)	48.51 (10.39)	50.10 (9.73)
	**40-54 years (n=49)**	49.53 (8.97)	47.18 (12.48)	45.56 (12.28)	49.44 (8.61)	51.48 (9.25)	49.58 (7.56)	49.68 (10.17)	46.67 (11.86)	46.79 (11.81)	46.84 (9.68)
	**≥55 years (n=15)**	46.73 (11.64)	51.41 (12.91)	42.51 (12.88)	47.66 (10.37)	52.01 (9.33)	50.53 (6.18)	48.42 (9.67)	50.89 (9.73)	47.51 (12.12)	50.72 (11.16)

*PCS* Physical health component summary *MCS* Mental health component summary *PF* Physical Functioning *RP* Role-Physical *BP* Bodily Pain.

*GH* General Health *VT* Vitality *SF* Social Functioning *RE* Role-Emotional *MH* Mental Health.

In total, 62 patients had a HADS-A score of 11 or greater, used in this study to suggesting a significant risk of clinically important anxiety and/or depression or “case status”. Four patients had a HADS-D score of 11 or greater, and all of those also had a high HADS-A score. There was no difference in the frequency of a significant HADS score in the under 25 years group compared with older patients (18.0% (11/61) vs 20.0% (51/254), χ2=0.12, p=0.73). Table 6 shows the results of a simple logistic regression models with a HADS-A or HADS-D score of 11 or greater as the dependent variable, adjusted for age and sex. Negative associations at under the 10% level of significance were noted between a clinically significant HADS score and being male, having a degree-level eduation, and being married. These associations were robust in a multivariable adjusted model (Table 7: model 1), where significant negative associations were again seen between having a clinically significant HAD score and being married or cohabiting vs single / divorced / widowed (Odds ratio (OR) 0.42, 95% confidence interval (CI) 0.19 to 0.92), and having education to third level or degree level (OR 0.31, 95% CI 0.14 to 0.71); with a trend towards significance for male sex (OR 0.54, 95% CI 0.29 to 1.01). There was a significant positive association between a clinically significant HADS score and being closely related to the family proband (OR 2.91, 95% CI 1.26 to 6.73).

**Table 6:** Simple logistic regression, with a significant HADS score as the outcome variable, with all models adjusted for age and sex

	**Odds ratio**	**95% confidence interval**	**P value**
Age (in years)	0.99	0.97, 1.01	0.41
Male sex	0.51	0.28, 0.91	0.02
First visit (vs subsequent)	0.95	0.49, 1.87	0.89
Degree level education vs lower	0.38	0.18, 0.83	0.014
Marital status: Married vs single/widowed / divorced	0.51	0.24, 1.07	0.074
Screening reason: SADS vs all other reasons	0.84	0.43, 1.65	0.61
Channelopathy vs all other reasons	0.94	0.49, 1.83	0.86
Cardiomyopathy vs all other reasons	1.21	0.69, 2.11	0.50
Family history of SCD	1.52	0.82, 2.80	0.18
First degree relative to family index case	2.24	1.06, 4.75	0.035

*SADS* Sudden arrhythmic death syndrome *SCD* Sudden cardiac death.

**Table 7:** Multiple regression models examining associations with a clinically significant HADS score

	**Model 1: Multiple logistic regression**			**Model 2: Multi level multiple logistic regression**		
	**Odds ratio**	**95% CI**	**P value**	**Odds ratio**	**95% CI**	**P value**
Male sex	0.54	0.29, 1.01	0.054	0.52	0.26, 1.03	0.062
Married (vs single/ divorced)	0.42	0.19, 0.92	0.031	0.40	0.17, 0.96	0.040
Third level education or higher	0.31	0.14, 0.71	0.006	0.31	0.13, 0.74	0.009
First degree relative to proband	2.91	1.26, 6.73	0.013	3.18	1.22, 8.31	0.018

Footnote. Multivariable models adjusted for age. Model 1 is a multivariable logistic regression, with a clinically significant HADS score as the outcome variable and adjusted for age. Model 2 is a multivariable logistic regression model with a random effects variable for family and adjusted for age. Log likelihood for model 1 is −130.45, and −130.05 for model 2. For the random effects model 2, the random intercept (ψ) is 0.253, and the estimated intraclass correlation of the latent responses (ρ) is 0.071.

Since patients in this study were undergoing their screening in a family context, the HADS and SF-12v2 scores were also evaluated by family, using a unique identification number for each family (n=135 families). For mean subscale score for the HADS and SF-12v2 subscales, between-family standard deviations were greater than within-family variations (Table 8). Finally, the effects of family on the regression model were examined using a random effects variable for the family unique identifier number (Table 7: model 2). Robust negative associations were observed between a clinically significant HADS score and the independent variables already described, although model fit as estimated by the log likelihood did not benefit from the addition of the random effects term.

**Table 8:** Between- and within- family effects on measures of psychological wellbeing and health-related quality of life, for 316 patients within 135 family groups

**Measure**	**Mean**	**Overall standard deviation**	**Between-family standard deviation**	**Within-family standard deviation**
***Hospital anxiety and depression scale***				
Anxiety scale	6.87	3.95	3.59	2.57
Depression scale	2.83	2.70	2.37	1.79
***Short form-12 Health-related quality of life***				
Physical health component summary	51.62	8.07	7.42	5.51
Mental health component summary	49.63	10.51	9.75	6.61

## Discussion

We describe high rates of significant psychological distress in our population of patients attending for family heart screening evaluation, with 19.2% of patients showing significant distress on their HADS scale. This distress was primarily related to high measures of anxiety. Younger persons also had high rates of significant HADS scores, with 18.2% of persons aged under 25 having significant distress on their HADS scale. However, HADS items relating to depression were more commonly endorsed in the older groups. We identified a number of parameters as significant predictors of greater distress at family screening evaluation. Patients who had lower levels of education, and who were not married, were more likely to have a significant HADS score, and there was a trend towards greater distress in female patients. Patients who were more closely related to the family proband were also at greater risk of a higher anxiety and depression score.

In our analysis, the particular screening indication (SADS vs channelopathy vs cardiomyopathy) was not associated with a difference in the health related quality of life (QoL) or psychological stress measures. This contrasts with another study, which noted poorer scores in the mental and physical health domains in patients attending for genetic screening for HCM, compared with those attending for genetic screening for arrhythmia or LQTS [14]. However this difference may be due to differences in the screening populations: our population were attending because of a family history and were mostly asymptomatic, whereas the other study population had a greater prevalence of both clinical HCM and symptoms. Female patients had a greater risk of a higher HAD score in our analysis, and a higher risk of heart-focused anxiety in women has been noted elsewhere also [23]. Girls who have been bereaved of a sibling are more at risk of higher grief and trauma scores than boys [24].

It is notable that a family history of SCD was not associated with higher HADS scores in our analysis. This is in keeping with the study by Christiaans and colleagues [25], where perceived risk of SCD was associated with psychological distress, but a family history of SCD was not. Conversely, another study has shown that using a different measure of distress, heart-focused levels of anxiety are higher in screening patients who have lost a close relative to sudden death [23]. We did not have data on the recency of the family bereavement (if any), and this may affect the level of distress encountered. However, being more closely related to the family index case or proband (whether alive or dead) was a positive associate of increased psychological distress in our analysis. Although it might be thought that SADS deaths might be most traumatic [24], there is no evidence that different causes of bereavement have different effects on grief and trauma reactions in young people [26]. We speculate that a closer relationship may mean that the patient had a better understanding of the event or illness which affected that person, and may attribute more importance to their screening evaluation. Such proximity may also mean that these patients might have been more directly affected by the proband’s illness or sudden death. These details may be teased out using a qualitative “family narrative” approach (personal communication, McGuinness S. Lived Lives lost to Suicide: A Visual Art Autopsy Study of Suicide in Ireland. PhD Thesis, UCD / NUI. December 2010).

There have been a number of investigations into the psychological well-being of patients with established cardiac diseases attending for cardio-genetic counselling [16,17,25,27,28] and/or specialist disease-related clinic services [14,29,30]. Studies have examined HADS findings in patients with conditions such as HCM [25,31] and DCM [32]. In these studies, both the HADS-A and HADS-D measures were higher than those seen in our screening group. In comparison, it has been shown that patients with a HCM genotype but with no clinical findings had better QoL measures that the general public, and were also less distressed than those gene carriers with structural cardiac changes [25]. However, these populations differ from the population described in this study, who were not known to have a cardiac condition at the time of their psychological testing. Attending for screening for potentially inheritable cardiac diseases has been shown to be associated with psychological stress. Hendricks et al. (2005) showed that parents of children undergoing genetic testing for LQTS have high levels of psychological distress at the time of testing, although levels of distress decrease gradually with time [33]. However the context of screening is important, with screening in the setting of no particular family history of cardiac diseases potentially less stressful. In one study, elite sportsmen screening was not associated with high levels of distress, with only 3% indicating significant stress on an Impact of Event Scale [15]. Furthermore, the potential personal impact of the screened condition may be relevant. In a group of patients attending for familial hypercholesterolaemia screening, with little change in QoL testing results either over time or between participants who had a positive (i.e. abnormal) finding versus those who did not carry the FH gene [17].

A new finding in our analysis was that variation in measures of psychological well-being was greater between families than within families. This is an important finding for clinicians involved in such screening evaluations. It may be that some families may be inherently predisposed to greater levels of anxiety and depression, or that the family diagnosis of SCD may be having a common effect on family members in certain families. In families with a bereavement, a complicated grief reaction in a number of family members is a predictor of clinical depression in the bereaved child or adolescent [34]. Should significant anxiety or depression be present in one family member, consideration should be given to assessing psychological well being in other members of that family also.

Our study presents data on a novel group of patients, with a high response rate. The two psychometric tests used showed good internal consistency and appropriate interscale correlation, and have been used in similar populations in the past, with the HADS score being previously validated in a HCM population [35]. Because of the established screening utility of the HADS, we used this measure as our outcome variable in the regression analyses. Dichotomising the outcome variable reduced the regression model information, but allowed us present odds ratios in a clinically useful way. The study was limited by a lack of contemporary comparator data, and the fact that the health-related QoL data were collected on one occasion only, with no follow up data available at this time. For the majority of patients, this was their first clinic visit, and they had not yet received patient education or genetic counselling or testing. Neither do we report on psychological status of patients attending after cascade genetic screening, as this population of patients is very small in Ireland at this time. Patients in Ireland typically access clinical cardiac screening in the first instance. We did not have complete information on variables such as time between the family SCD (for those families in which a SCD had occured) and the screening visit, nor did we have information on who had discovered the deceased person. As very small numbers of patients were on any medical therapy (such as beta blockers), no conclusions could be drawn as to the putative effects of same on psychological wellbeing.

These findings have clear implications for cardiac family-based screening practice. Clinicans should be aware of the potential for significant psychological distress in screening patients, and not alone in those with a family history of a SCD or SADS bereavement. Closer attention should be afforded families where one member has elevated anxiety, as it is more likely that elevated anxiety will be present in other family members. From our clinical observation, we believe that patients’ understanding and interpretation of family risk may be quite different to that of clinicians, and clinicians should take this variability into account when conducting screening evaluations. Assessing patient knowledge, and confronting specific concerns through patient education, is important for this vulnerable group.There is an established rationale for a multidisciplinary approach to high-risk cardiac screening, with inputs from multiple health care stakeholders, and the role of the specialist cardiac screening nurse and the cardiac genetic counsellor has been endorsed by other authorities [1,2,16].

## Conclusions

High levels of anxiety were seen amongst patients attending a family-based cardiac screening clinic, even amongst younger patients, and measures of psychological wellbeing and QoL showed less variance within families than between families.

## Competing interests

The authors declare that they have no competing interests.

## Authors’ contributions

CMcG, NGM, JON, JG and MC designed the study. CMcG and CMcS undertook study recruitment, and database and study document design. CMcG, CMcS and CMcQ performed the data analysis and interpretation. CMcG, CMcS & CMcQ drafted the manuscript, with all other co-authors providing comments. All co-authors had access to anonymised study data, and CMcG takes responsibility for the integrity of the data and the accuracy of the data analysis. All authors read and approved the final manuscript.

## Pre-publication history

The pre-publication history for this paper can be accessed here:

http://www.biomedcentral.com/1471-2350/14/1/prepub
